# Intestinal intraepithelial lymphocyte activation promotes innate antiviral resistance

**DOI:** 10.1038/ncomms8090

**Published:** 2015-05-19

**Authors:** Mahima Swamy, Lucie Abeler-Dörner, James Chettle, Tanel Mahlakõiv, Delphine Goubau, Probir Chakravarty, George Ramsay, Caetano Reis e Sousa, Peter Staeheli, Barbara A. Blacklaws, Jonathan L. Heeney, Adrian C. Hayday

**Affiliations:** 1 Immunosurveillance lab, Francis Crick Institute, Lincoln's Inn Fields Laboratories, London WC2A 3LY, UK; 2 Peter Gorer Department of Immunobiology, King's College London, Borough Wing, Guy's Hospital, Great Maze Pond, London SE1 9RT, UK; 3 Cell Signalling and Immunology, College of Life Sciences, University of Dundee, Dow Street, Dundee DD1 5EH, UK; 4 Department of Veterinary Medicine, University of Cambridge, Cambridge CB3 0ES, UK; 5 Institute of Virology, University Medical Center, Freiburg D-79104, Germany; 6 Spemann Graduate School of Biology and Medicine, Albert Ludwigs University Freiburg, Freiburg, Germany

## Abstract

Unrelenting environmental challenges to the gut epithelium place particular demands on the local immune system. In this context, intestinal intraepithelial lymphocytes (IEL) compose a large, highly conserved T cell compartment, hypothesized to provide a first line of defence via cytolysis of dysregulated intestinal epithelial cells (IEC) and cytokine-mediated re-growth of healthy IEC. Here we show that one of the most conspicuous impacts of activated IEL on IEC is the functional upregulation of antiviral interferon (IFN)-responsive genes, mediated by the collective actions of IFNs with other cytokines. Indeed, IEL activation *in vivo* rapidly provoked type I/III IFN receptor-dependent upregulation of IFN-responsive genes in the villus epithelium. Consistent with this, activated IEL mediators protected cells against virus infection *in vitro*, and pre-activation of IEL *in vivo* profoundly limited norovirus infection. Hence, intraepithelial T cell activation offers an overt means to promote the innate antiviral potential of the intestinal epithelium.

## Abstract (web summary)

Intraepithelial lymphocytes in the gut eliminate dysfunctional epithelial cells and promote regrowth of healthy cells. Here the authors show that, in addition, these lymphocytes protect cells against viral infections by rapidly activating interferon-dependent pathways in intestinal epithelial cells.

## Graphical abstract

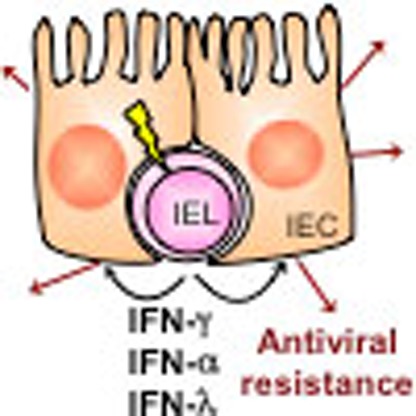

Intraepithelial lymphocytes (IEL) compose a very large T cell compartment conserved in all vertebrates. Thus, intestinal IEL exist from fish through mice to humans. Despite their conservation, and their decades'-old description, IEL remain very poorly understood, particularly in humans^1^. This situation cannot sustain, given the cells' juxtaposition to the intestinal microbiota that are increasingly acknowledged to regulate many aspects of health and disease. Thus, there is a growing pressure to use animal models to clarify the diversity and basic biology of IEL.

Murine small intestinal IEL comprise roughly equal numbers of γδ and αβ T cells. One type of IEL (previously termed Type *a*) expresses T cell antigen receptor (TCR)αβ and CD8αβ and comprises mostly Tissue-resident memory (T_RM_) cells recruited to the epithelium after systemic priming^1^,^2^. The second type of IEL (Type *b*) expresses either TCRαβ or TCRγδ, and is either CD8αα^+^ or CD8^−^CD4^−^ (so-called double negative cells). Type *b* IEL are the more common type in unchallenged mice and seemingly enter the small intestinal epithelium directly upon maturation, rather than experiencing a systemic phase^3^. Furthermore, many are considered to be agonist selected and reactive to auto-antigens, although there is scant direct evidence for this^4^.

Both IEL sub-compartments contain cells that can be rapidly activated via TCR-dependent and/or TCR-independent stimulation, permitting them to influence the afferent phase of an immune response rather than solely the delayed efferent phase—the traditional arena of T cell responses^1^,^5^. Many studies have focused on the potent cytotoxicity of activated IEL^6^, their promotion of epithelial repair^7^, and their capacity to recruit other immune-protective and immune-regulatory cells. Indeed, within hours of activation, skin and/or gut IEL can produce very large amounts of biologically active soluble mediators, including granzymes, cytokines and chemotactic factors^6^,^7^,^8^. Conversely, the potential of IEL to contribute key, anti-pathogen responses has not been so clear.

Given the plethora of bacteria in the gut and its potential to regulate T cell composition and function^9^,^10^,^11^, there has predictably been interest in the role of IEL in anti-microbial responses. Thus, recently reported work, consistent with the contribution of TCRγδ^+^ IEL to the immediate phase of immune responses, demonstrated the cells' capacity to limit bacterial dissemination during the first few hours following infection with *Salmonella enterica* ser. Typhimurium. This activity was mainly dependent on cell-intrinsic production of the antibacterial defensin, RegIIIγ^12^. Likewise, earlier work demonstrated both immunoregulatory and immunoprotective roles for murine IEL in the response to the natural and ubiquitous gut-tropic infectious protozoan parasite, *Eimeria*^13^,^14^,^15^.

Enteroviruses, including coxsackieviruses, rotavirus and norovirus, cause debilitating disease in neonates, but often much milder disease in adults, a phenomenon frequently attributed to the relative immaturity of the neonatal gut immune compartment^16^. Of note, IEL numbers are low at birth and during weaning, but expand substantially thereafter, coinciding with the reduced susceptibility of adult mice to multiple viruses^4^,^17^,^18^. Added to this, IEL expand after systemic infection with lymphocytic choriomeningitis virus^19^ and during oral infection by reovirus and rotavirus^20^,^21^. Within 48 h of infection, interferon-γ (IFNγ) production was increased, as was the cells' cytotoxic activity. Moreover, the cytotoxic activity of primed IEL specifically targeted virus-infected cells in culture^22^. Collectively, these findings strongly argued for the further investigation of how IEL activation might promote virus-resistance within the epithelium.

To accomplish this, we have employed two strategies. First, a recently developed protocol for culturing IEL-permitted IEL–intestinal epithelial cell (IEC) interactions to be studied *in vitro*. Second, these interactions were validated *in vivo* by examining the molecular and biological impact upon IEC of IEL activated by the intraperitoneal (i.p.) administration of anti-CD3. Unexpectedly, one of the most conspicuous impacts of activated IEL upon IEC was the upregulation of IFN-stimulated genes (ISGs) and of the antiviral response, attributable to the production of type I, II and III IFNs by IEL. Consistent with this, activated IEL supernatants rendered epithelial cells more resistant to virus infection *in vitro*. Moreover, T cell activation *in vivo* rapidly induced ISGs in the villus epithelium in mice, corresponding with greatly enhanced resistance to murine norovirus (MNV) within 40 h of gastrointestinal infection. By identifying a novel and powerful potential for intestinal T lymphocytes to regulate the innate antiviral defences of the surrounding stroma, these findings underline the innate-like biology of the IEL compartment and its importance for body surface integrity.

## Results

### IEL activate antiviral responses in epithelial cells

To examine how soluble effectors produced by activated IEL might impact upon IEC, we prepared microarray gene expression profiles of the widely used, murine small intestinal epithelial cell line MODE-K after treating it for 18 h with supernatants of IEL that had been exposed for 24 h either to agonistic TCR cross-linking or to control antibodies. Because IEL rapidly die *ex vivo*, particularly when challenged with TCR agonists, activated IEL supernatants are invariably contaminated with products of stressed and dying cells. To avoid this, a recently developed culture system (ref. 23 and see Methods) was employed that maintains IEL in a highly viable state for over 2 weeks, permitting them to be grown out and rested before re-activation. Relative to the starting preparation, the cultures at 13 days post-establishment comprised essentially pure T cells (Supplementary Fig. 1a,b), removing, for the first time, the possibility that mediators attributed to IEL might be the products of contaminating cell types. The cultures largely preserved the subset composition of the γδ T cell compartment (Supplementary Fig. 1a,b), thereby permitting assay of type *b* IEL. The subset complexity of TCRαβ^+^ IEL, comprising both type *a* and type *b* cells was also preserved, albeit that the cells' relative representation changed over time (Supplementary Fig. 1a,b).

**Figure 1:**
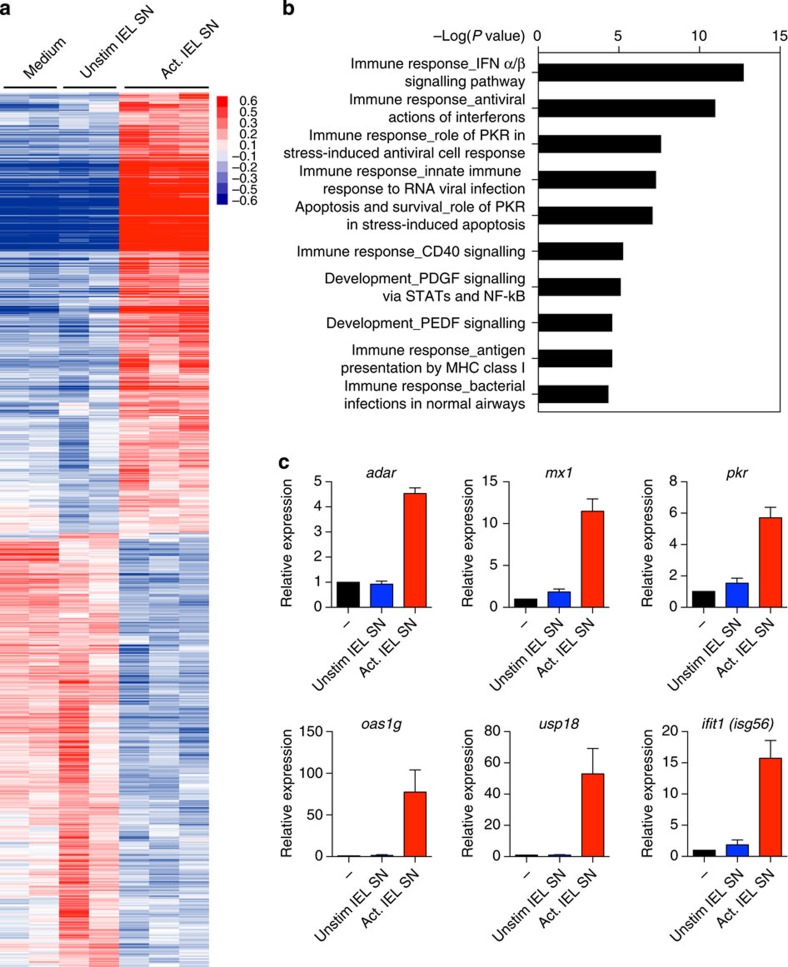
IEL supernatant (SN) induces antiviral defence pathways in IEC. (**a**) Hierarchical clustering of differentially expressed genes in MODE-K cells treated with SN from unstimulated cultured IEL and SN from IEL activated on plate-bound anti-CD3 for 24 h, in comparison to genes expressed in MODE-K cultured in medium alone. Heatmap key: red=higher expression, blue=lower expression relative to the mean expression of the gene across all samples. (**b**) Genes upregulated in MODE-K by stimulated IEL SN relative to unstimulated IEL SN were subjected to enrichment analysis using Metacore. Shown are the top ten statistically significant pathways, calculated using an adjusted *P* value of 0.05. (**c**) Confirmation by qRT–PCR of upregulation of some antiviral genes. MODE-K cells were cultured for 18 h with medium, anti-CD3, or SN from cultured IEL as in **a**. Data are the mean and s.e.m. of three to five independent experiments calculated as fold change relative to the untreated samples. See also Supplementary Fig. 1.

The IEL were re-activated by exposure to plate-bound anti-CD3 (ref. 23) following which supernatants were collected. RNA expression profiles from MODE-K cells treated with activated and resting IEL supernatants from several biologically independent cultures were analysed and compared on an Illumina platform. By comparison to medium alone or to supernatant from unstimulated IEL, the supernatant of anti-CD3-activated IEL profoundly impacted upon MODE-K cells, with many genes overtly upregulated, and others strongly downregulated (Fig. 1a). This study focuses on the unexpected finding that 22 of the 50 most upregulated genes related to antiviral functions (Table 1). Indeed, Metacore Genego pathway enrichment analysis showed that the top four pathways upregulated comprised IFN-regulated genes associated with antiviral activity (Fig. 1b), including *Mx2, Rsad2* (Viperin), multiple *Oas* genes*, Ifit3* and *Bst2* (Tetherin; Table 1).

The upregulation of ISGs in MODE-K cells cultured either with IEL supernatant or with IEL in the presence of anti-CD3 was confirmed by quantitative reverse transcription–PCR (qRT–PCR), extending the ISGs analysed to other well-characterized examples, for example, *Ifit1, Mx1, Adar, Oas1g* and *Pkr (eif2ak2)* (Fig. 1c). Although some other ISGs, for example, *Ifih1 (MDA5)* and *Ifitm1/2* were not upregulated, it is known that different cell types stimulated with IFNs under different circumstances induce discrete sub-groups within the full spectrum of ISGs^24^,^25^,^26^.

IEL supernatants were initially applied to MODE-K cells for 18 h. However, subsequent experiments showed that some ISGs were upregulated within 6 h, strongly suggesting them to be direct responses to activated IEL effectors (Supplementary Fig. 1c). Moreover, increases in expression could be achieved with supernatants from IEL that had been activated for just 2–3 h, implying that the IEL mediators responsible for the induced antiviral signatures in epithelial cells were produced immediately upon anti-CD3 stimulation (Supplementary Fig. 1d). Last and notably, both TCRαβ^+^ and TCRγδ^+^ IEL could evoke this response in MODE-K cells, as stimulation of essentially pure, IEL-derived T cell cultures (Supplementary Fig. 1b) with either anti-TCRβ or anti-TCRγδ antibodies induced the IEC response signature (Supplementary Fig. 1e).

### Activated IEL upregulate type I and III IFNs

**Figure 2:**
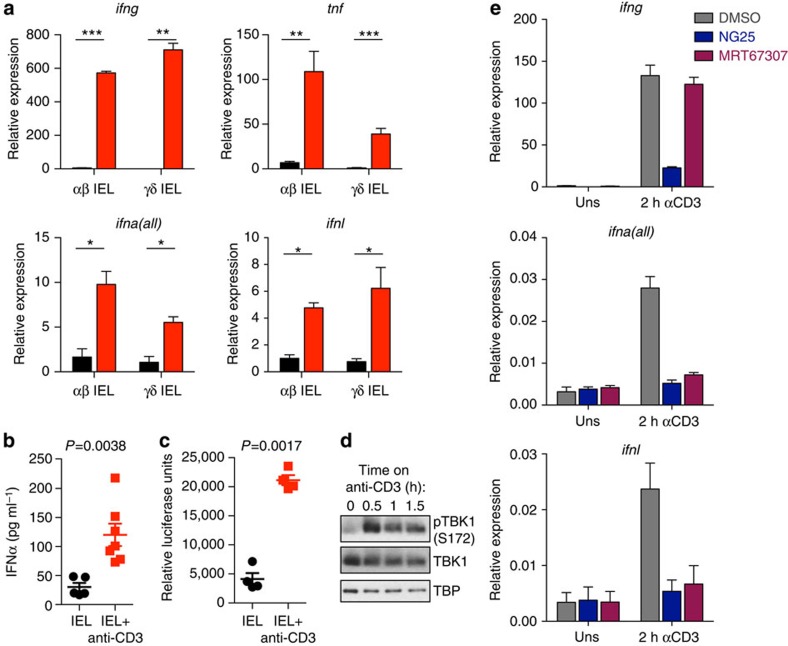
IEL produce type I and III IFN upon stimulation through activation of TBK1. (**a**) qRT–PCR showing expression of type I, II, III IFN mRNA and TNF mRNA in IEL sorted into TCRγδ^+^ CD8αα^+^ and TCRαβ^+^ CD8αα^+^ subsets, cultured and then stimulated with anti-CD3 for 2 h (red bars). Data are the mean and s.e.m. of IEL from three mice. (**b**) IFNα was measured in resting and activated (18 h) IEL (cultured) supernatant by ELISA. Each point represents an independent culture, *n*=7. (**c**) IEL supernatant was assayed for the presence of active IFN by ISRE-luciferase reporter assay, *n*=4. Statistical significance was calculated in **a**–**c** by two-tailed unpaired *t*-tests, *P<0.05, **P<0.01, ***P<0.001. (**d**) Cultured γδTCR^+^ IEL were stimulated on plate-bound anti-CD3. The cells were then lysed and analysed by western blotting for phosphorylation of TBK1 (S172), total TBK1 and the loading control TBP (full blots are shown in Supplementary Fig. 2d). Data are representative of four independent cultures. (**e**) IEL were pre-treated with 1 μM of each of the following inhibitors for 1 h (NG-25 (TAK1 inhibitor) and MRT67307 (TBK1/IKKɛ inhibitor)), before stimulation on anti-CD3 for 2 h. mRNA for IFNα, IFNλ and IFNγ were measured by qRT–PCR (Uns=unstimulated). Mean and s.e.m. of four technical replicates are shown, and the data represent two independent experiments. DMSO, dimethylsulphoxide.

ISGs are induced by IFNs, primarily type I IFN (including IFN-α/β) and type III IFN (IFN-λ2, λ3, also known as interleukin (IL)-28a,b in the mouse)^24^,^25^,^26^. To test whether activated TCRγδ^+^ and/or TCRαβ^+^ IEL produce IFNs, IEL were this time flow-sorted *ex vivo* to substantially pre-enrich for either TCRαβ^+^ or TCRγδ^+^ CD8αα IEL (Supplementary Fig. 2a), and then cultured for 12–14 days before being stimulated for 2 h by anti-CD3 stimulation. Although the composition of the sorted cells changed somewhat in culture, the respective subsets remained greatly enriched for TCRαβ^+^ IEL and γδ IEL (Supplementary Fig. 2b), and in each case, substantial increases in *Ifna* mRNA were provoked by TCR agonist stimulation (Fig. 2a). *Ifnl* mRNA was also rapidly upregulated albeit to a lesser extent than known products of IEL activation such as tumour necrosis factor (TNF) and IFNγ (Fig. 2a).

These results were consistent with the finding (above) that either anti-TCRβ or anti-TCRγδ antibody treatment of IEL cultures induced the IEC response signature. We therefore checked that similar responses would be induced in IEL that were flow-sorted directly *ex vivo*, briefly rested, and then activated before any culturing. Indeed, they too upregulated transcription of *Ifna, Ifnl* and *Ifng* mRNAs (Supplementary Fig. 2c).

Of note, supernatants of cultured IEL that had been activated for 18 h accumulated IFNα sufficient to be easily measurable by ELISA (Fig. 2b), and to trigger luciferase expression in cells transfected with an ISRE-containing promoter responsive to type I and type III IFNs (Fig. 2c). Thus, TCR-mediated activation of IEL can rapidly trigger functionally competent type I and type III IFNs, in addition to its induction of conventional T effector cytokines.

TCR-triggered production of type I IFNs has been previously documented in CD8^+^ T cells^27^, but the pathway leading to this induction was not elucidated. The transcription of type I and type III IFNs is generally induced by Interferon Regulatory Factors, IRF3 and IRF7 (refs 28, 29), contingent on their phosphorylation by TBK1/IKKɛ that promotes their nuclear translocation and transcriptional targeting^30^. Consistent with their expression of type I and type III IFNs, IEL constitutively express *Irf7* mRNA, which encodes a limiting transcriptional regulator of IFN production (Supplementary Fig. 2c; Serial analysis of gene expression data, gene expression omnibus (GEO) accession code GSE63090 (ref. 6)), while TCR triggering of resting IEL induced phosphorylation of TBK1/IKKɛ on the activation loop Ser172 (Fig. 2d and Supplementary Fig. 2d). Furthermore, the induction after 2 h of *Ifna* and *Ifnl* mRNAs was clearly reduced by inhibition of IKKɛ/TBK1 by MRT67307 (ref. 31), whereas this had little effect on *Ifng* upregulation, which was instead reduced by NG-25 that inhibits TAK1 (albeit with some impact on Lck; Fig. 2e). The fact that NG-25 also inhibited *Ifna* and *Ifnl* mRNA induction is consistent with TCR-dependent TAK1-mediated phosphorylation of TBK1/IKK. Collectively, these data offer a basic molecular mechanism by which type I and III IFNs may be induced in IEL directly by TCR triggering.

The co-expression by activated IEL of type I, II and III IFNs and TNF is provocative given that these may cooperatively and variably regulate different gene sets. For example, blocking the type I IFN receptor (IFNAR-1) on MODE-K cells during culture with IEL supernatant substantially impaired induction of *Mx1, Usp18* and other ISGs (Supplementary Fig. 3a), whereas the blocking antibody did not affect *Irf1*, which is known to be induced very strongly by IFNγ and not by IFNα (ref. 24; Supplementary Fig. 3a). Indeed, upregulation of each gene tested was reduced to baseline levels by simultaneously blocking the response to more than one IFN/cytokine. We did not use blocking antibodies against IFNlambda receptor (IFNLR, IL28R), as MODE-K cells do not express mRNA for this receptor (Supplementary Fig. 3b). However, because IFNλ produced by IEL may also contribute *in vivo* to ISG upregulation in primary epithelial cells expressing IFNLR, the studies described below continued to focus on both type I IFN and IFNλ (type III IFN).

### Activated IEL protect epithelial cells from virus infection

**Figure 3:**
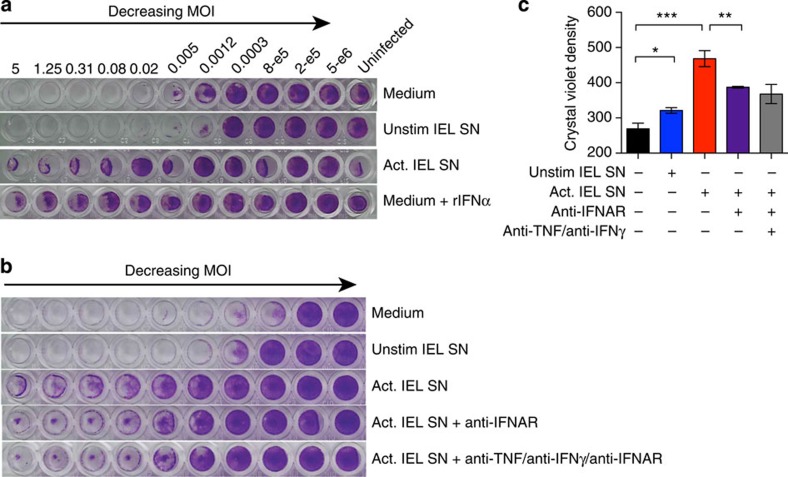
IEL supernatant (SN) protects epithelial cells from virus infection in culture. (**a**) MODE-K cells were infected with decreasing concentrations of EMCV (encephalomyocarditis virus). Starting with an MOI of 5 (3 × 10^5^ virus particles (leftmost)) a serial fourfold dilution was made along the row. Before infection SN of unstimulated or activated cultured IEL was added to the indicated rows. Recombinant IFNα (500 U ml^−1^) was added as a control. (**b**) A similar experiment was performed as in **a**, but in addition to adding IEL SN to MODE-K cells before infection, blocking antibodies against IFNAR1 and/or IFNγ and TNF were added to IEL SN and then added to the MODE-K cells. Data are representative of three or six rows for each condition in **a** and **b**. (**c**) Quantification of data in **b**, significance was calculated by two-tailed unpaired *t*-test with Welch's correction, *P<0.05, **P<0.01, ***P<0.001.

If activated IEL supernatant is a source of type I IFN and IFNλ, we would predict that it should protect cells from virus infection. To test this, supernatant of cultured IEL, with or without TCR-mediated stimulation (Act or Unstim) was added to MODE-K cell cultures that were then infected with encephalomyocarditis virus (EMCV), which induces a cytopathic effect in many murine cell types. As indicated by crystal violet staining of living cells (Fig. 3a,b), EMCV efficiently killed MODE-K cells even at low multiplicity of infection (MOI). This cytopathic effect was reduced by adding anti-CD3-activated IEL supernatant to MODE-K cells before infection, whereas supernatants from resting IEL had no appreciable effect (Fig. 3c). The protective effect was roughly comparable to that of 500 U ml^−l^ of recombinant IFNα, showing the potency of IEL activation in protecting epithelial cells from virus. It was partially blocked by antibodies to the type I IFN receptor (IFNAR-1), or by blocking the three effectors, type I IFN, IFNγ and TNF together (Fig. 3b,c). Thus, in the absence of any additional cell types, soluble mediators produced by activated IEL increased the antiviral resistance of IEC.

### IEL activation *in vivo* induces a rapid antiviral response

**Figure 4:**
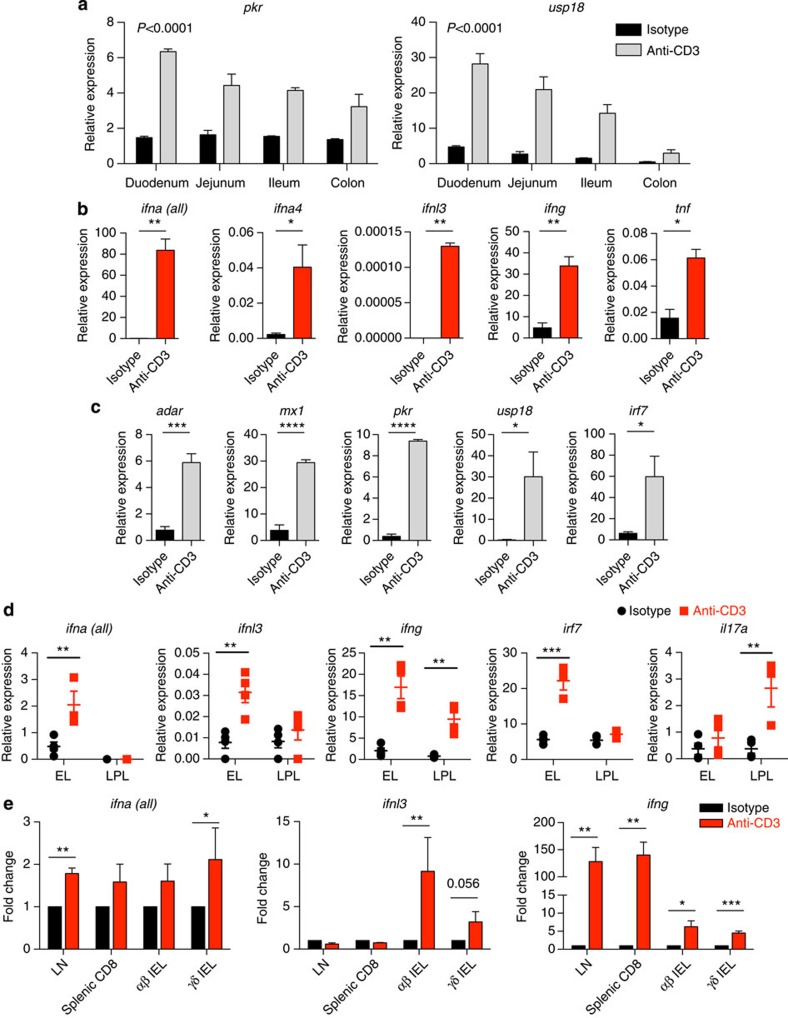
Upregulation of ISGs occurs rapidly *in vivo* upon T cell activation in the intestine. (**a**) WT mice were injected i.p. with control IgG (isotype) or 50 μg of anti-CD3. The intestines were harvested 3 h post injection, and cDNA prepared from small sections of the indicated regions: qRT–PCR was used to measure the upregulation of ISGs as before. Error bars indicate s.e.m. of three mice per condition. Shown are the *P* values for anti-CD3 to isotype comparison, calculated by two-way analysis of variance. (**b**,**c**) The epithelial cell layer was harvested from small intestines of mice treated as in **a** and then sorted on a MACS column for CD45-positive (mainly IEL (**b**)) and CD45-negative (mainly intestinal epithelial cells (**c**)) fractions: qRT–PCR was performed as before. Expression is shown relative to TBP expression. Mean and s.e.m. of *n*=3 and the data shown are representative of four independent experiments. (**d**) Mice (*n*=4) were injected i.p. with 25 μg anti-CD3 antibody or isotype, and after 3 h, the epithelial layer (EL) and LP layer (LPL) were extracted, and analysed by qRT–PCR as in **b**. (**e**) Mice were injected i.p. with 25 μg anti-CD3 antibody or isotype, and after 3 h the inguinal lymph nodes (LNs), spleen and IEL were isolated. TCRγδ^+^ CD8αα^+^ and TCRαβ^+^ CD8αα^+^ IEL were flow-sorted, and analysed by qRT–PCR, alongside sorted CD8^+^ cells from spleens and total LN cells. Normalized fold change from four experiments is shown. Statistical significance was determined by either unpaired *t*-tests (**b**,**c**,**e**) or using the Holm-Sidak method (**d**). *P<0.05, **P<0.01, ***P<0.001. Flow cytometry of sorted cells and histology sections are in Supplementary Fig. 4.

Although the data to this point were derived by use of the MODE-K cell line, they clearly provoked the hypothesis that IEL activation induces antiviral response genes in neighbouring epithelial cells *in vivo*. To test this, a single low dose of anti-CD3 was administered i.p. Relative to mice receiving isotype control antibodies, antiviral genes were clearly rapidly upregulated (within 3 h; Fig. 4a), with a gradient of induction broadly correlating with IEL density along the length of the small intestine^32^. By fractionating total epithelium from the small intestine, the induction of *Ifna, Ifnl, Ifng* and *Tnf* could be attributable to the CD45^+^ fraction (mainly CD8α^+^ TCRαβ^+^ and TCRγδ^+^ IEL, see Supplementary Fig. 4a), whereas the antiviral genes were upregulated in the CD45^−^ fraction (∼80% EpCAM^+^ IEC, Supplementary Fig. 4a and Fig. 4b,c). Peak induction for some genes was at 3 h, with some mRNAs returning to baseline by 6 h (Supplementary Fig. 4b,c), emphasizing how rapid are the effects of activated T cells on epithelial cells *in vivo*.

At the 3 h time point at which gene expression was assayed, there was no major damage to the epithelial layer and no major immune cell infiltration (Supplementary Fig. 4d,e). However, because there was some villus blunting reminiscent of intestinal inflammation^33^, it was appropriate to consider other possible lymphocyte sources of T cell activation-dependent *Ifn* gene expression. Thus, i.p. injection of anti-CD3 was repeated in WT mice and 3 h later the epithelial and lamina propria (LP) compartments separately isolated from the same guts (flow cytometry data of isolated IEL are shown in Supplementary Fig. 4f,g). qRT–PCR on the extracted layers clearly showed that IFNα and IFNλ gene upregulation almost exclusively segregated with IEL, which also showed signatory high IFNγ RNA induction (Fig. 4d). Conversely, the LP fraction displayed preferential induction of *Il17a*, a known signature of LP lymphocytes, but negligible *Ifna* upregulation, and very weak *Ifnl* upregulation. Thus, there was no basis for conjecturing that intestinal T cell-dependent induction of anti-viral effectors *in vivo* reflected the activity of LP T cells.

Also at 3 h after TCR triggering *in vivo*, we isolated splenic T cells, inguinal lymph nodes (LNs) and IEL that were flow-sorted into the main unconventional T cell populations (TCRαβ^+^ CD8αα^+^ IEL and TCRγδ^+^ CD8αα^+^ IEL; Supplementary Fig. 4h). Splenic CD8 T cells and LN cells upregulated *Ifna* RNA to some extent, consistent with previous reports^27^, and *Ifng* mRNA very strongly, but they did not upregulate *Ifnl* RNA. Conversely, only purified IEL factions showed reproducible upregulation of RNAs encoding all IFN subtypes (Fig. 4e). (The relative attenuation of the type I IFN response probably reflects the fact that this is only a transiently induced gene-set relative to the time (∼7 h) required for harvesting tissue and flow cytometry-based cell sorting before RNA preparation.) Interestingly, the mild villus blunting apparent by histology (Supplementary Fig. 4d,e) has previously been associated with IFNα treatment of mice^34^, possibly reflecting anti-CD3 induced type I and type III IFN expression by IEL.

### ISG upregulation *in vivo* depends on IFNα and IFNλ receptors

**Figure 5:**
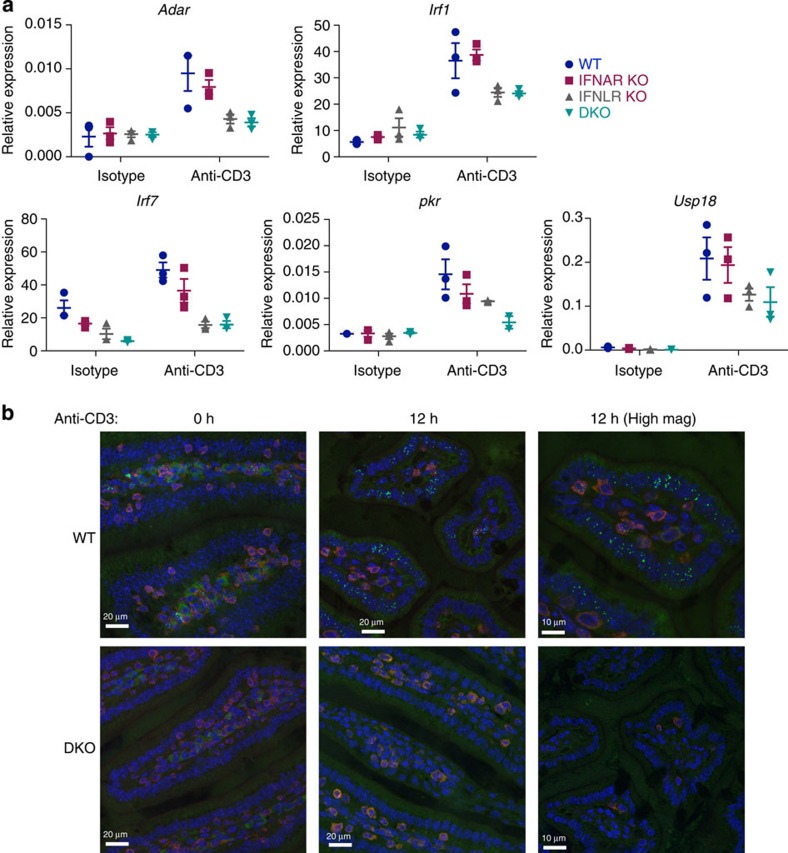
Induction of ISGs depends on both type I and III IFNs *in vivo*. (**a**) B6.A2G-Mx1 wild type (WT), IFNAR KO, IFNLR KO and IFNAR/IFNLR DKO mice were treated with 25 μg anti-CD3 or isotype control (ITC) intra-peritoneally, and intestinal tissue sections prepared after 3 h. Induction of mRNA of the indicated ISGs was measured by qRT–PCR in wt, IFNAR KO, IFNLR KO and IFNAR/IFNLR DKO mice in small intestinal tissue, and expression relative to TBP is shown. Each data point represents an individual mouse. (**b**) Tissue sections from anti-CD3 i.p. injected B6.A2G-Mx1 wt and IFNAR/IFNLR DKO mice were prepared at 0 h and 12 h after injection. Paraffin-embedded sections were stained for Mx1 protein (intra-nuclear, green), CD3 (red) and nuclei (4,6-diamidino-2-phenylindole, blue). Pictures are representative of three mice for each condition. Scale bar, 20 μm. Also shown are micrographs at higher magnifications (High mag) taken at 12 h p.i., showing the nuclear speckle-like localization of Mx1 in the wt mouse gut, but not in the IFNAR/IFNLR DKO mouse gut. See also Supplementary Fig. 5.

To test the contribution of type I and type III IFNs to ISG upregulation *in vivo*, anti-CD3 was administered i.p. to mice mutant for one or both IFN receptor subtypes. The levels of induction of many ISGs were reduced in *Ifnar1*^*−/−*^ mice, commonly to a greater degree in *Ifnlr1*^*−/−*^ mice, and most often to an even greater degree in *Ifnar1*^*−/−*^*Ifnlr1*^*−/−*^ double knockout (DKO) mice (Fig. 5a). Of note, the rapid induction of *Irf1*, which depends primarily on IFNγ (see Supplementary Fig. 3a), was little impaired in *Ifnar1*^*−/−*^*Ifnlr1*^*−/−*^ DKO mice.

To check if antiviral protein was induced by T cell activation *in vivo*, a C57BL/6 strain was employed that harbours functional alleles of *Mx1*, which is a pseudogene in wild–type C57BL/6 mice^35^. Mx1 protein rapidly accumulates in the nucleus following induction via type I or type III IFN receptors but not via IFNγR^36^,^37^. Under steady-state conditions, a well-characterized antibody against Mx1 (refs 37, 38) detected the specific punctate nuclear stain for Mx1 protein in a very small number of LP cells (Fig. 5b and Supplementary Fig. 5a; note that the homogeneous cytoplasmic stain seen in some LP cells reflects a background signal seen also in some Mx1-deficient mice, Supplementary Fig. 5b). Conspicuously, the punctate nuclear pattern of Mx1 protein was overtly upregulated in isolated or clustered cells throughout the epithelium, consistent with an ongoing IFN response (Fig. 5b and Supplementary Fig. 5a). This activation-induced expression pattern was not seen in intestines of *Ifnar1*^*−/−*^*Ifnlr1*^*−/−*^ DKO mice after anti-CD3 stimulation (Fig. 5b and Supplementary Fig. 5a), establishing that its TCR-driven upregulation depended on type I/type III IFN.

### Pre-activation of IEL reduces susceptibility to norovirus infection

MNV is an enteric non-enveloped positive-sense, single-stranded RNA virus (family calicivirus) naturally prevalent among mice^39^,^40^. Multiple strains of this virus have been isolated that cause either acute or persistent asymptomatic infection in immunocompetent mice^39^,^41^. Mice are infected by the faecal-oral route with evidence for infection of gastrointestinal epithelial cells, macrophages and dendritic cells^39^,^42^. The capability to grow MNV in macrophage cell lines and then to infect its natural host make it an important animal model for infection by caliciviruses, including human norovirus. Furthermore, the growth of MNV is greatly inhibited by IFNs, particularly IFNλ (ref. 43).

**Figure 6:**
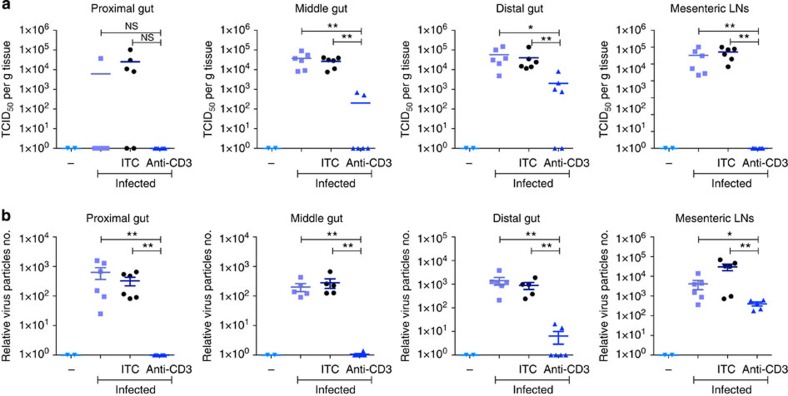
Activation of IEL is protective against murine norovirus (MNV) *in vivo*. C57BL/6 mice were orally infected with MNV-O7, without pre-treatment (no label), or 8 h after treating mice i.p. with anti-CD3 antibodies or the isotype control IgG (ITC). 40 h after infection, the organs were isolated and assayed for live virus (TCID_50_) (**a**) or analysed by qRT–PCR for the presence of viral RNA (**b**). Uninfected mice were used as controls (−). Virus copy numbers were estimated based on a standard curve, and then normalized for the amount of tissue section taken by normalizing to expression of a house-keeping gene (TBP) (**b**). Each point represents an individual animal, and statistical significance between columns was determined by two-tailed unpaired *t*-test (Kolmogorov–Smirnov method). *P<0.05, **P<0.01. See also Supplementary Fig. 6.

Therefore, to test whether prior TCR-dependent activation in the gut might increase epithelial resistance to norovirus, mice were infected orally with MNV-O7 (ref. 44) 8 h after pre-inoculation with either control or agonistic anti-CD3 antibodies i.p. After 40 h (a time point chosen to ensure detection of virus-infected cells), the intestines and mesenteric LNs from infected and uninfected controls were isolated, and levels of virus replication assessed by live virus titration (TCID_50_ assay; Fig. 6a and Supplementary Fig. 6a), and by qRT–PCR for viral RNA (ORF2; Fig. 6b and Supplementary Fig. 6b), expressed in each case on a logarithmic scale. Notwithstanding some variation between groups, live virus was readily detected by TCID_50_ assay in cells harvested from infected mice or from infected mice pre-treated with an isotype-control antibody, with consistent results obtained by qRT–PCR. However, when measured by either assay, the level of infection was reproducibly and substantively decreased in independent experiments in which mice were pre-treated with anti-CD3 (Fig. 6). Even in one case where the reduction in anti-CD3 pre-treated mice was not statistically significant (in proximal small intestine, as measured by the TCID_50_ assay (Fig. 6a)), it was noteworthy that no virus could be detected in those mice (0/6), by comparison to the detection of virus in the small intestines of 5 out of 12 mice of the control groups, and that significance was reached when the same tissue was assayed by reverse transcription–PCR (Fig. 6b). Consistent results were obtained whether virus copy number was assessed relative to a control gene (Fig. 6b) or to mass of tissue (Supplementary Fig. 6b). Note too, that the results are not consistent with large amounts of virus in anti-CD3-treated mice translocating from the intestine into systemic compartments, as the viral load was also dramatically reduced (between 10-fold and 100-fold) in the mesenteric LNs, consistent with there being a severe overall attenuation of infectious virus (Fig. 6a). Thus, TCR-dependent IEL activation *in vivo* functionally enhances innate resistance to norovirus.

## Discussion

The very clear finding of this study is that upon activation, intestinal intraepithelial T cells can functionally promote innate antiviral immunity in neighbouring cells thereby contributing to timely host protection. Two questions immediately arise. The first question is the type of scenario in which IEL will be activated via the TCR. The IEL compartment is one of the largest in the body and broadly conserved across vertebrates. Data presented in this study show that multiple types of intestinal IEL can invoke antiviral effects. This is consistent with two recent studies of T_RM_ cells, although neither study explored the functional potential of T_RM_ cells beyond their production of conventional T cell mediators, particularly IFNγ and TNF^45^,^46^. Type *a* IEL will likely include virus specific, memory CD8^+^ T_RM_ cells, deposited in the epithelium following prior infection^47^,^48^,^49^,^50^, and rapidly re-activated via the presentation of viral peptides by infected epithelial cells. Moreover, there may be biological utility to bacteria-/parasite-specific IEL activating anti-viral immunity, given recent work showing how gastrointestinal bacteria may facilitate virus infection^51^,^52^.

The physiologic TCR stimuli for Type *b* IEL have not been determined, but they are widely considered to include self-antigens that reflect cellular stress or dysregulation that is a consequence of infection. Such TCR-mediated responses can be aided by detection of ‘stress-ligands' for the activating NKG2D receptor that is particularly strongly expressed by human intestinal IEL. Indeed, NKG2D is such an important component of anti-viral T- and NK cell responses that widely diverged viruses have each developed distinct immuno-evasive mechanisms^53^. In addition, IEL can respond to IL-15 that can be produced in high amounts by infected IEC^54^,^55^. Hence, via responses to antigen, NKG2D ligands, and ‘cytokine alarmins', Type *b* IEL may contribute to the innate phase of antiviral immunity via their sensing of the status of the tissue^5^. In so doing, the antiviral effects of IEL activation would complement other impacts of IEL on IEC survival, differentiation and turnover^56^,^57^: the aggregate effect being to eradicate potentially dangerous infected epithelial cells; to promote repair of an intact epithelium; and to reduce susceptibility to further virus infection. By these several means, T cells can play a key role in the early phase of the response to challenge, an arena ordinarily assigned to innate myeloid cells.

The second question would ask whether activated IEL (and possibly other T cells) are over-looked, inducible sources of IFNs. Of note, the anatomical restriction of IFNλ production to the epithelial layer of the intestine fits with the restriction of the IFNλ receptor expression to epithelial cells and barrier surfaces. Moreover, IEL express *Irf7* that encodes the major transcriptional regulator of type I and type III IFNs. IRF7 is itself a target of the type I IFN response, indicating that IEL themselves are probably responding to low levels of IFNs that they constitutively produce. Of note, a study by Ogasawara *et al*. of splenic CD8^+^ T cells also noted the cells' autocrine response to their own low levels of type I IFN^27^. We have now indicated roles for the key kinases upstream of IRF7 activity, TBK1 and/or IKKɛ, upon TCR-triggering of IEL. Furthermore, the *Ifnb1* and *Ifnl3* promoters also contain nuclear factor-κB-responsive elements, potentially providing an additional link of TCR triggering to the induction of IFNs^29^,^58^.

The impact of activated IEL supernatants on upregulating antiviral effectors in epithelial cells is rapid, most likely direct, and inhibited *in vitro* and *in vivo* by blocking or removing type I and type III IFN receptors. As noted previously, not all known ISGs were upregulated in epithelial cells. This may partially depend on the cohort of signal transducers and activators of transcription factors that are activated downstream of the respective IFN receptors, as has been shown for the differential response of lymphocyte subsets to IFNβ^59^. However, this study also emphasizes that the antiviral effect is promoted by a collaboration of type I and/or type III IFN with type II IFN and TNF that collectively comprise signature products of IEL. This collaboration can occur at multiple levels, involving receptor cross-talk at the level of the membrane, and further downstream, through the signalling complexes involved^60^.

Clearly, our work should not be taken to suggest that IEL are the exclusive source of IFNs for IEC. Rather, IEL may be important sources of IFNs in response to antigens and/or to upregulated NKG2D ligands, neither of which is sensed by myeloid cells. Interestingly, earlier studies showed that IEC express the type I IFN receptor apically and not basolaterally, and suggested that this would make IEC unresponsive to type I IFNs produced by other cells^61^. Consistent with this, Mx1 induction in IEC after rotavirus infection *in vivo* was more largely dependent on type III IFNs than on type I IFNs^61^. Nonetheless, given that IEL extend processes upwards between IEC, as do dendritic cells, it may be that apical IFNAR is specifically responsive to IFNs produced locally by IEL.

It is also the case that the impact of IFNs and the genes that they induce is not limited to antiviral functions. For example, protein kinase, dsRNA-activated (PKR), which phosphorylates the translation initiation factor eIF2α, modulates several stress-related signalling pathways including autophagy by halting translation^62^. Likewise, IFN-responsive transporter associated with antigen processing-1 (TAP1) is required for major histocompatibility complex-I antigen presentation in addition to its more recently discovered antiviral attributes^26^, and several IFN-inducible GTPases have antimicrobial activity^63^. Moreover, guanylate-binding protein-1 upregulation in IEC strengthens barrier integrity by reducing epithelial apoptosis^64^. Thus, IEL activation may promote such biological activities relevant to body surface integrity and epithelial turnover.

In sum, this study demonstrates an overlooked role for tissue-associated T cells in promoting the antiviral innate defences of the surrounding tissue. This in turn shows that a fundamental component of innate immunity can be regulated by cells classically regarded as adaptive cells, re-emphasizing caution in studies of innate immunity undertaken in scenarios of adaptive immune dysfunction, such as recombinase activating gene (RAG)-deficient mice or paediatric lymphocyte deficiencies. Finally, there is increasing public health concern about gastrointestinal virus infection in the developing and the developed worlds^65^,^66^. In considering the basis for resistance to such infections, it would seem important to consider potential contributions of T cells, as considered here. Indeed, T cell production of type I and III IFNs might usefully be considered in a similar clinical context to T cell production of cytokines such as IL-17 and IFNγ; that is to say, a potential route to immune enhancement for vaccination strategies and a target for suppression in the case of autoimmunity, inflammation or graft rejection.

## Methods

### Mice

Six- to twelve-week-old female C57BL/6 mice were obtained from Charles River Laboratories (Harlow). B6.A2G-Mx1 wild type mice carrying intact Mx1 alleles (WT), B6.A2G-*Mx1-Ifnar1*^*−/−*^ mice lacking functional type I IFN receptors (IFNAR knockout (KO)), B6.A2G-*Mx1-Ifnlr1*^*−/−*^ mice lacking functional type III IFN receptors (IFNLR KO) and B6.A2G-*Mx1-Ifnar1*^*−/−*^*Ifnlr1*^*−/−*^ mice (DKO) lacking functional receptors for type I and III IFN were bred at the animal facility at the University of Freiburg. Animals were housed in accordance with the guidelines defined by the Federation for Laboratory Animal Science Associations (http://www.felasa.eu/recommendations) and experiments were performed in compliance with the German animal protection law (TierSchG) and the Animal (Scientific Procedures) Act UK (1986) and approved by the local animal welfare committees of the Universities of King's College London, Cambridge, Freiburg and Dundee.

### Cell lines

The MODE-K small intestinal enterocyte cell line was a kind gift of D. Kaiserlain (U. Lyon, France)^67^. LL171 cells (L929 cells containing a stable IFN-stimulated response element-luciferase reporter plasmid (ISRE-Luc)) were a kind gift from Mireia Pelegrin (Montpellier, France).

### Isolation and culture of murine small intestinal IEL

IEL were isolated from mouse small intestine as previously described^23^. Briefly, the small intestines were opened, freed of Peyer's patches and washed in PBS. The epithelial layer was gently scraped off using a scalpel, and incubated for 40 min in RPMI 1640 containing 10% FCS and 1 mM dithiothreitol in a turning wheel. After centrifugation and vortexing in RPMI, the cells were passed through a nylon wool column. Isolated cells were centrifuged in a 20%/40%/80% Percoll density gradient at 700*g* for 30 min. The IEL were harvested from the 40 to 80% Percoll interface. After isolation, IEL were stimulated on plate-bound anti-CD3 antibody (1 μg ml^−l^, 145-2C11, BD, BioLegend) in a cocktail of cytokines (IL-2 (10 U ml^−1^), IL-15 (100 ng ml^−1^) (Immunotools), IL-3 (100 U ml^−1^), IL-4 (200 U ml^−1^) (Roche)). After 2 days, cells were transferred to fresh plates and cultured with IL-2 (10 U ml^−1^) in RPMI 1640 Supplemented with 10% FCS, 100 U ml^−1^ penicillin, 100 μg ml^−1^ streptomycin, 2 mM glutamine, 1 mM sodium pyruvate, 1 mM β-mercaptoethanol, 2.5 mM HEPES, and non-essential amino acids (all from Invitrogen). Cells were maintained in 96-well round-bottom plates at a concentration of 500,000 cells per ml (200 μl per well) at 37 °C and 10% CO_2_. Medium was replaced completely every 3–4 days.

### IEL activation and blocking antibodies

For activation and experiments, IEL were seeded into 96-well round-bottom plates (70,000–100,000 cells per well in 200 μl) pre-coated with antibodies against CD3 (145-2C11, BioLegend). In blocking experiments, IEL supernatants were pre-incubated with antibodies against TNF (TN3–19), IFNγ (R4-6A2; eBioscience), anti-IFNAR (BioLegend). Recombinant cytokines were purchased from Peprotech.

### *In vivo* IEL activation

C57BL/6 female mice aged 8–12 weeks were intraperitoneally injected with 25 or 50 μg anti-CD3ɛ antibody (145–2C11) or an isotype control (ITC) antibody (both BioLegend, low endotoxin, azide-free) and killed after 3 h. The intestine was excised and flushed with PBS. Samples from different gut regions were taken for immunohistochemistry and RNA analysis. The remaining small intestine was cut longitudinally, Peyer's patches excised and cut into 1 cm pieces and incubated for 30 min in RPMI 1640 containing 10% FCS and 1 mM dithiothreitol at room temperature in a turning wheel. Cells were spun down, resuspended in RPMI 10% FCS and vortexed for 4 min. The released cells were passed through a 70-μm cell strainer, the remaining tissue was collected and again vortexed in RPMI 10% FCS for 4 min. The released cells were pooled and centrifuged at 700 × *g* for 30 min (no brake) in a density gradient of 80/40/20% Percoll. Purified IEL and IEC fractions were either directly taken for flow cytometric analysis, sorting, or further purified by CD45 magnetic bead columns (Miltenyi). Purified cells were used for RNA analysis. The tissue remaining after IEL extraction was then subjected to a further extraction of the LP layer by digesting the tissue with Collagenase D (500 μg ml^−1^) and DNase (50 μg ml^−1^) for a further 1 h at 37 °C. The tissue was mashed through a 70-μm filter, and the resulting cells also purified on a 40% Percoll layer.

### Microarray analysis

MODE-K cells were seeded into six-well plates and cultured overnight. Medium was replaced with supernatant from unstimulated IEL, supernatant from anti-CD3-stimulated IEL (100% of assay volume), or medium containing recombinant TNF and IL-1α (10 ng ml^−1^ and 100 pg ml^−1^, respectively). RNA was isolated using the Qiagen RNeasy extraction kit, labelled by the standard Illumina biotin labelling protocol and analysed on Illumina gene expression arrays (Mouse Ref. 8 v3) at the Bart's and the London Genome Centre. Gene expression data were analysed using Bioconductor 2.2 (http://bioconductor.org) running on R2.7.1. Normalized probe-set expression measures were calculated using log2 transformation and quantile normalization using the ‘Lumi' package. To determine significant differences of expression levels between the different groups, a moderated Student's *t*-test was computed on a gene-by-gene basis using the empirical Bayes statistics in the ‘Limma' package. The resultant *P*-values were adjusted for multiple testing using the false discovery rate (FDR) Benjamini and Hochberg method, where any probe sets that exhibited an adjusted *P*-value FDR *q*<0.05 were called differentially expressed. Cluster was used to draw the heatmap using average linkage clustering to cluster the genes. Differentially expressed probes were also analysed using Metacore Pathway analysis tool (Thomson Reuters) to identify enrichment of pathways and processes. The analysis employs a hypergeometric distribution to determine the most enriched gene set. Pathways or processes that showed FDR *q*<0.05 were called as enriched.

### Viruses, stimuli, and cytokine induction assays

EMCV strain pEC9 was a gift from Dr Ian Kerr. MODE-K cells were plated at 30,000 cells per well in 96-well flat-bottom plates. The cells were infected with decreasing concentrations of EMCV starting with an MOI of 100 (3 × 10^5^ virus particles (leftmost)) then a serial fourfold dilution was made along the row. One column of cells was left uninfected to control for cell proliferation and viability. Where indicated, the cells were pre-treated overnight with 1:1 diluted IEL supernatant, with/without blocking antibodies. Four rows were prepared for each treatment. Twenty-four hours post infection, cells remaining in the well were fixed and stained with crystal violet. A clear well indicates cell death by virus infection. To quantify the crystal violet data, the 8-bit images of 96-well plates were analysed by measuring mean density with background density subtracted in each well. For stimulation and cytokine induction assays, MODE-K cells were seeded in 48-well plates at 4 × 10^5^ cells per ml and treated with IEL supernatant or recombinant cytokines for 6 h unless otherwise indicated.

### IFNα detection

IFNα was measured by sandwich ELISA as described previously^68^. Total IFNα/β were measured by titration on LL171 (ISRE-Luciferase containing) cells and compared with recombinant human IFN-A/D used as a cytokine standard.

### Quantitative real-time reverse transcription–PCR

Samples were stored in RNAlater (Ambion) or directly frozen in RLT buffer, RNA was isolated with a Qiagen RNeasy kit, transcribed into cDNA and analysed in comparison to TATA box binding protein (TBP) on a ViiA7 Real-time PCR machine (Applied Biosystems). All primer sequences are provided in Supplementary Table 1.

### Immunohistochemistry

Gut tissue was fixed overnight in 10% neutral-buffered formalin, paraffin embedded and sectioned at 4-μm thickness for staining with mouse anti-Mx1 (clone M143)^38^ or anti-CD3ɛ (rabbit polyclonal, Dako). Secondary antibodies used were AlexaFluor 488- and 647-labelled antibodies from Molecular Probes (Invitrogen). Sections were mounted in Vectashield containing 4,6-diamidino-2-phenylindole to stain nuclei.

### Imaging

Images were acquired at room temperature on a Zeiss-LSM710 confocal microscope with a Plan-Apochromat × 40/1.3 Oil/DIC objective and Zen image acquisition software or on a Leica SP5 laser-scanning confocal microscope equipped with a × 40/1.4 numerical aperture PL APO oil objective. All images were acquired as z-stacks of 10 μm thickness and maximum projections presented using the Leica application suite software. Images were exported and processed using Volocity software (Improvision) or ImageJ.

### *In vivo* virus infection

The MNV strain O7 (MNV-O7) was isolated from the faeces of a *STAT1*^*−/−*^ mouse, grown in BV2 cells^69^ and used at passage 3. The strain establishes a persistent, and usually non-pathogenic, infection in immunocompetent mice^44^. Six- to eight-week-old female C57BL/6 mice were injected i.p. with either 25 μg anti-CD3ɛ antibody (145-2C11) or isotype control antibody or were left untreated. After 8 h, mice were then infected with 5 × 10^6^ TCID_50_ MNV-O7 by oral gavage and killed at 40 h post-infection. Tissues were removed immediately and the intestine flushed through with PBS before sectioning. Sections were preserved in RNAlater for qRT–PCR or were frozen at −80 °C for assays to titre live virus. Sections were weighed, homogenized in PBS, freeze-thawed, supernatant clarified and the resulting supernatant used in limiting dilution infection assays. Live virus titre was calculated by the Reed-Muench method^70^.

### Statistics

Statistical analyses were performed using Prism 6.0 (GraphPad), and the tests used are indicated in the figure legends. Differences were considered significant when *P*<0.05.

## Additional information

**How to cite this article:** Swamy, M. *et al*. Intestinal intraepithelial lymphocyte activation promotes innate antiviral resistance. *Nat. Commun.* 6:7090 doi: 10.1038/ncomms8090 (2015).

## Supplementary Material

Supplementary Information Supplementary Figures 1-6, Supplementary Table 1

## Figures and Tables

**Table 1:** Top 50 genes upregulated by activated IEL supernatant activity in MODE-K cells, relative to treatment with unstimulated IEL supernatant*.
