# Supplementary material for: Intestinal intraepithelial lymphocyte activation promotes innate antiviral resistance
Source: Nat Commun. 2015 May 19;6:7090. doi: 10.1038/ncomms8090 (PMC4479038; doi:10.1038/ncomms8090)
Supplement: Supplementary Information — Supplementary Figures 1-6, Supplementary Table 1 [file ncomms8090-s1.pdf]

Supplementary Figure 1 (to Fig. 1, Swamy et al)

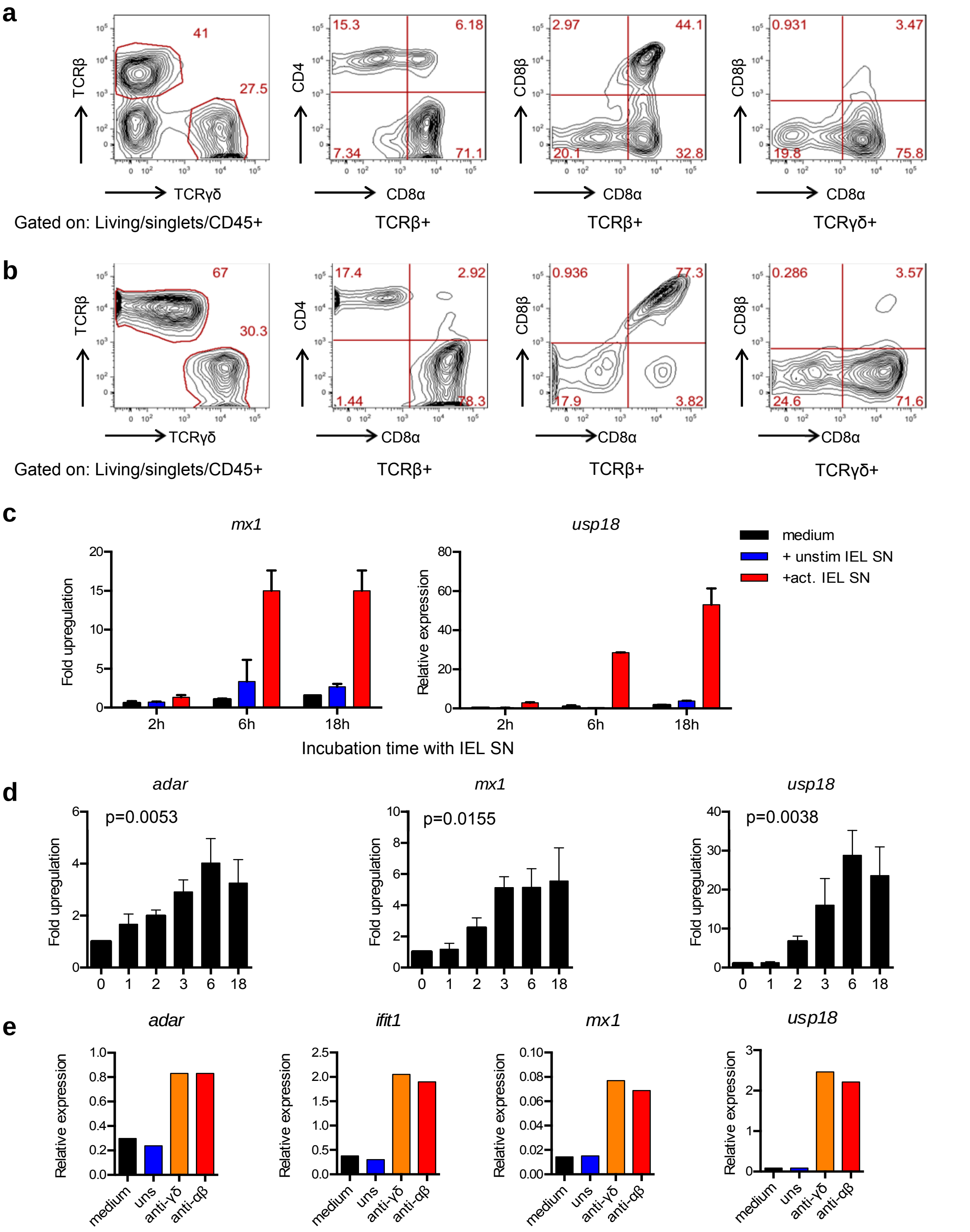

Supplementary figure 1. IEL rapidly produce ISG-inducing activity upon anti-CD3 stimulation. IEL include TCRαβ^{+}CD8αβ^{+}, TCRαβ$^{\square}$CD8αα^{+}, TCRγδ^{+}CD8αα^{+}, and TCRγδ^{+} DN populations, found directly *ex vivo* (a), and after 13 days culture (b): living CD45^{+} cells before and after 13 days of culture were stained for TCRβ and TCRγδ (left-hand panel), and the

CD8α, CD8β and CD4 phenotypes determined for TCRβ^{+} cells (middle-panels) and TCRγδ^{+} cells (right-hand panel). (c) Upregulation of ISGs as measured by qRT-PCR relative to TBP, in MODE-K cells. MODE-K cells were treated for the indicated number of hours with IEL supernatant (SN) from cultured IEL re-stimulated on anti-CD3 for 18h. Shown is a representative experiment. (d) Cultured IEL were stimulated on plate-bound anti-CD3 for the indicated number of hours, and the supernatant used to treat MODE-K for 6h. qRT-PCR was performed and 3 experiments were normalized to untreated controls. Data shows the mean and SEM of the fold upregulation of 3 independent experiments. p-values were calculated by Kruskal-Wallis tests. (d) 12-day cultured IEL were unstimulated (uns) or re-stimulated on plate-bound anti-TCRαβ (H57) or anti-TCRγδ (GL3) for 18 hours and supernatant harvested. The supernatants were used to treat MODE-K cells for 6h, and cDNA prepared from the treated MODE-K cells. qRT-PCR depicting expression of ISGs relative to TBP is shown.

Supplementary Figure 2 (to Fig. 2, Swamy et al)

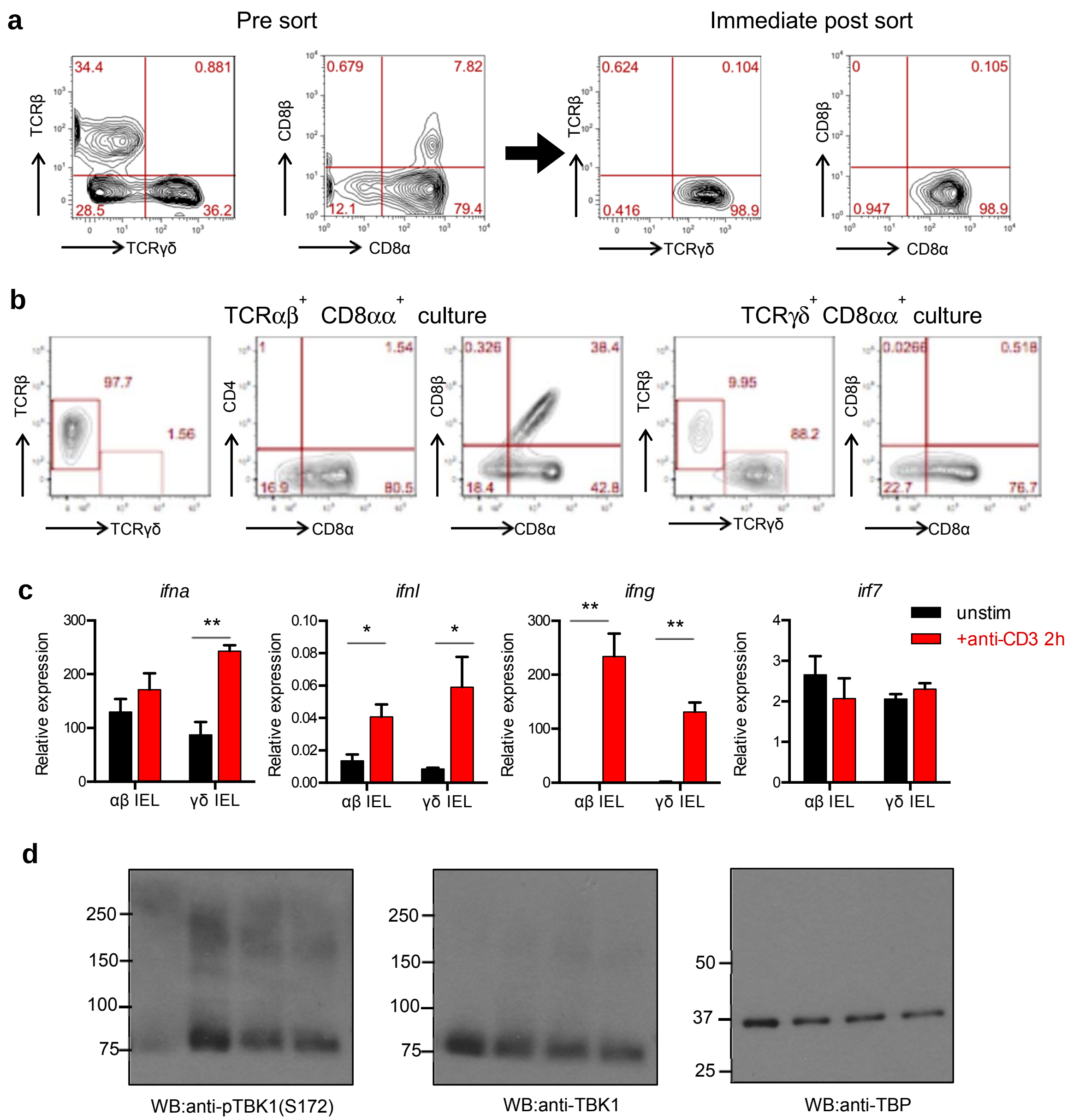

Supplementary figure 2. *Ex vivo* IEL upregulate IFNα, IFNλ and IFNγ mRNA upon stimulation. (a) Representative flow cytometry data of sorted TCRγδ$^+$ CD8αα$^+$ IEL, shown pre- and immediately post-sort. Sorted TCRγδ$^+$ and TCRβ$^+$ IEL were then directly stimulated with anti-CD3 (data are shown in Suppl. Fig. 2c) or were cultured for two weeks and then re-assessed by flow cytometry (b), prior to stimulation with anti-CD3 (for data shown in Fig. 2a). (c) qRT-PCR showing expression of Type I, II, III IFN mRNA and *irf7* mRNA in IEL flow-sorted

immediately *ex vivo* into TCRγδ$^+$ CD8αα$^+$ and TCRαβ$^+$ CD8αα$^+$ subsets, briefly rested, and then stimulated with anti-CD3 for 2 hours (red bars), without prior culture. Data are the mean and SEM of IEL from 3 mice. (d) Full images for western blots shown in Fig. 2d. Molecular weight markers in kDa are indicated to the left of each blot.

Supplementary Figure 3

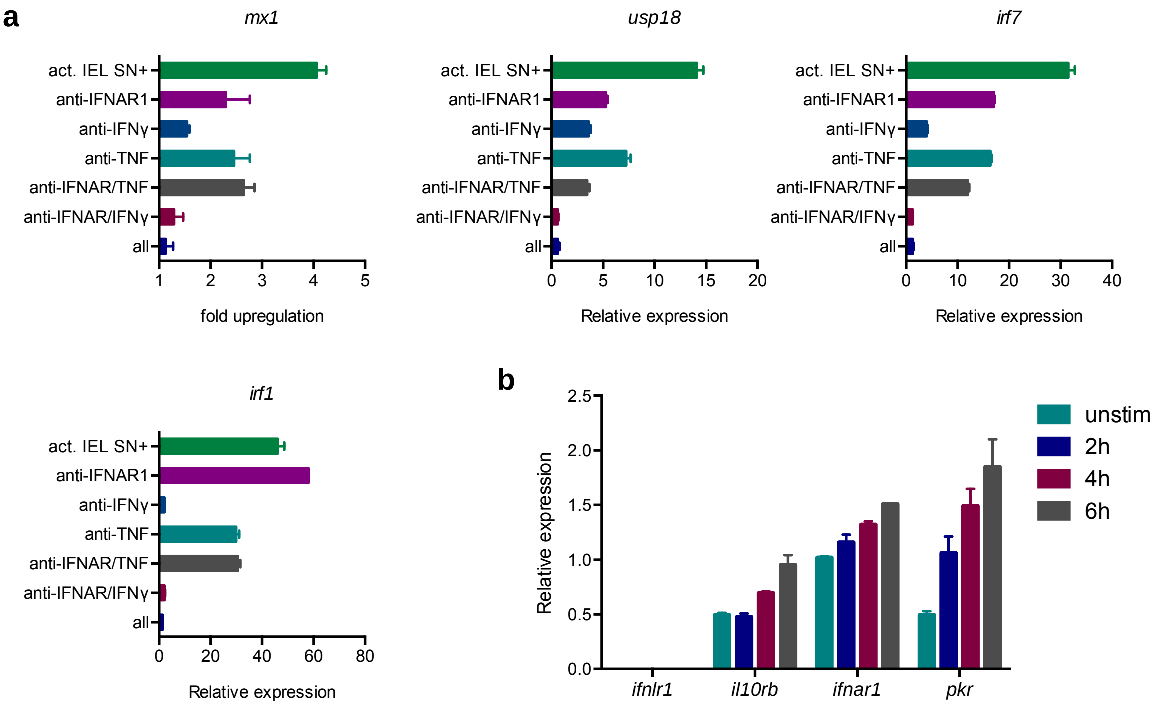

Supplementary figure 3. Upregulation of antiviral genes involves multiple cytokines produced by IEL. (a) MODE-K cells were treated for 6 hours with activated IEL supernatant (6 hours) to which neutralizing/blocking antibodies against IFNγ (5µg/ml), TNF (1µg/ml) and/or IFNAR1 (1µg/ml) had been added alone or in combinations. cDNA was prepared and upregulation of ISGs measured by qRT-PCR. Data are means + SEM of triplicate wells normalized to medium control, and are representative of $\geq$ 3 independent experiments. (b) Cultured IEL were stimulated on plate-bound anti-CD3 for the indicated number of hours, and the supernatant used to treat MODE-K for 6h. qRT-PCR was used to measure the indicated gene expression in MODE-K. Whereas *il10rb* (co-receptor for IFNλ), *ifnar1* and *pkr* mRNA were easily detected, mRNA for *ifnlr1* was not expressed.

Supplementary Figure 4 (to Fig. 4, Swamy et al)

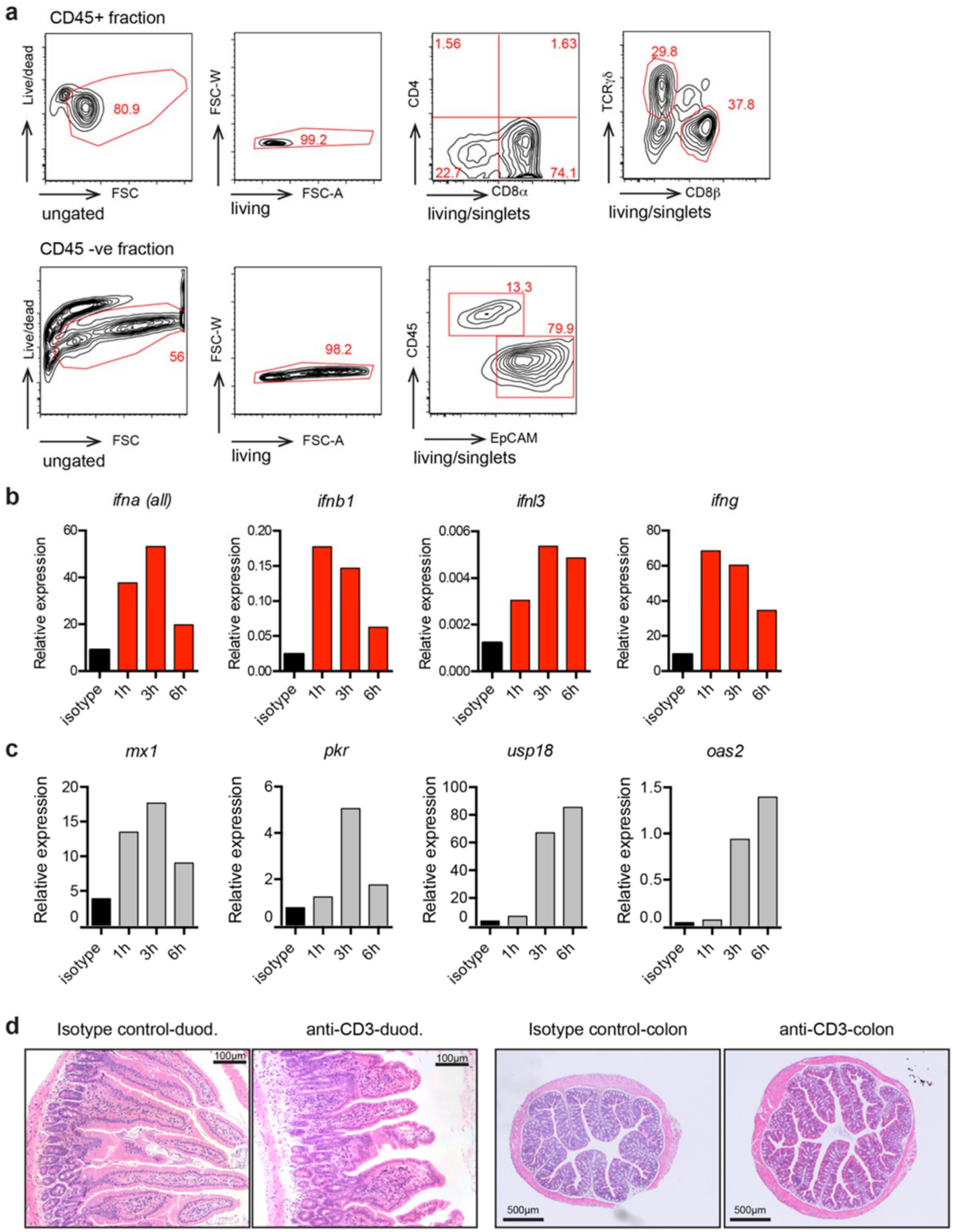

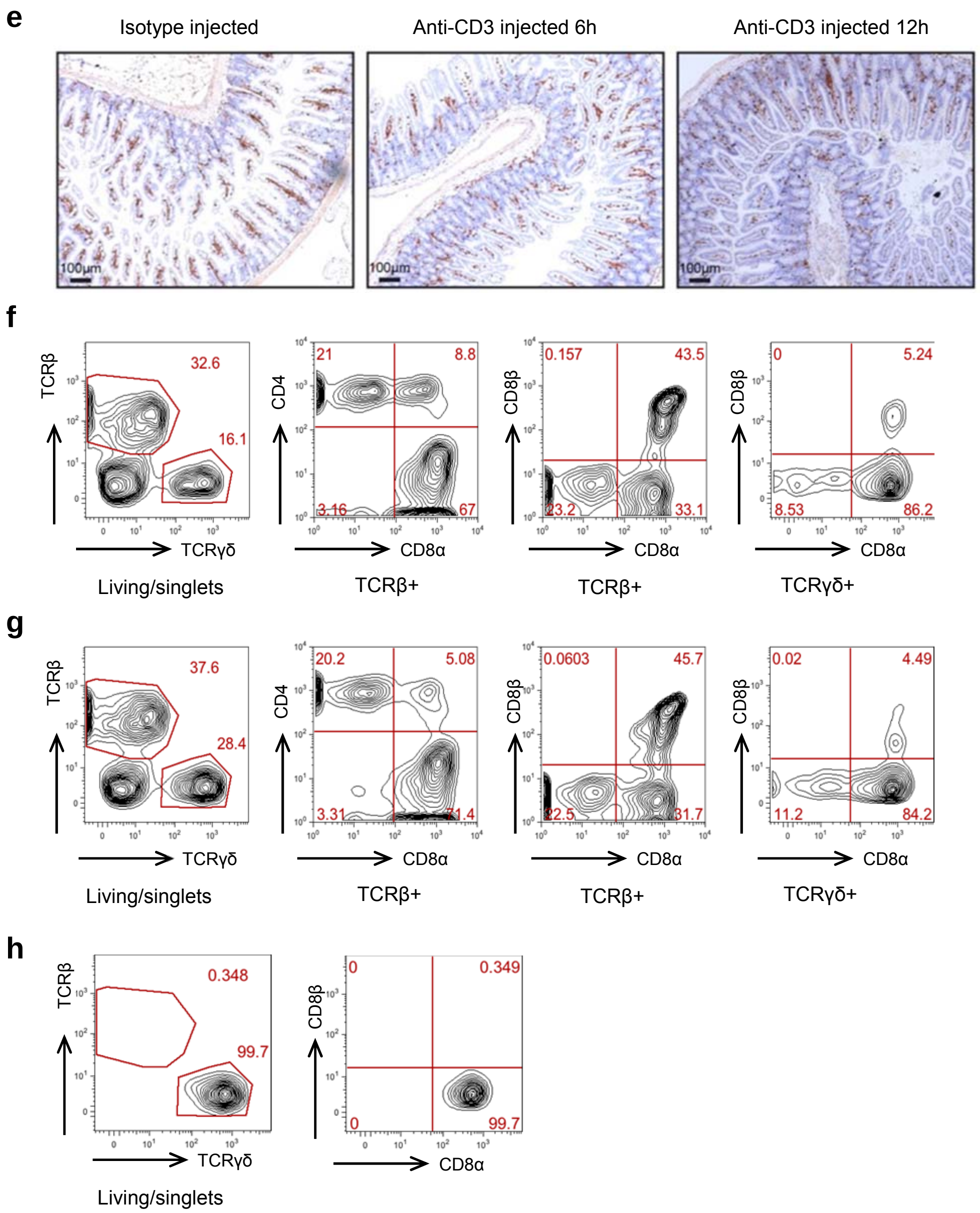

Supplementary Figure 4. Anti-CD3 i.p. injection does not cause major damage to the epithelium yet induces rapid upregulation of ISGs. (a) Representative flow cytometry of $CD45^{+}$ and $CD45^{-}$ MACS-sorted cells used in Figure 5b and 5c. (b, c) C57BL/6 mice were injected i.p. with 25μg anti-CD3 antibody and mice sacrificed after 1, 3 or 6 hours, respectively. 25μg Armenian hamster IgG was injected into the control, which was sacrificed after 3h (isotype). IEL (b) and IEC (c) were isolated from small intestines by Percoll gradient; RNA was prepared and samples were analysed by qRT-PCR. Gene expression is shown relative to

expression of TBP. (d) H&E stained duodenal and colonic sections taken 3h after anti-CD3 or isotype injection. (e) Anti-CD3 stained (brown) sections of intestinal tissue from isotype-injected (12h) and anti-CD3 injected (6h and 12h) mice. (f-h) Flow cytometry data showing cells isolated 3h after i.p. injection of anti-CD3 (f) or isotype control (g). (h) purity of sorted TCRγδ CD8αα cells from an anti-CD3 injected mouse used for the analyses shown in Fig. 4e.

Supplementary Figure 5 (to Figure 5, Swamy et al)

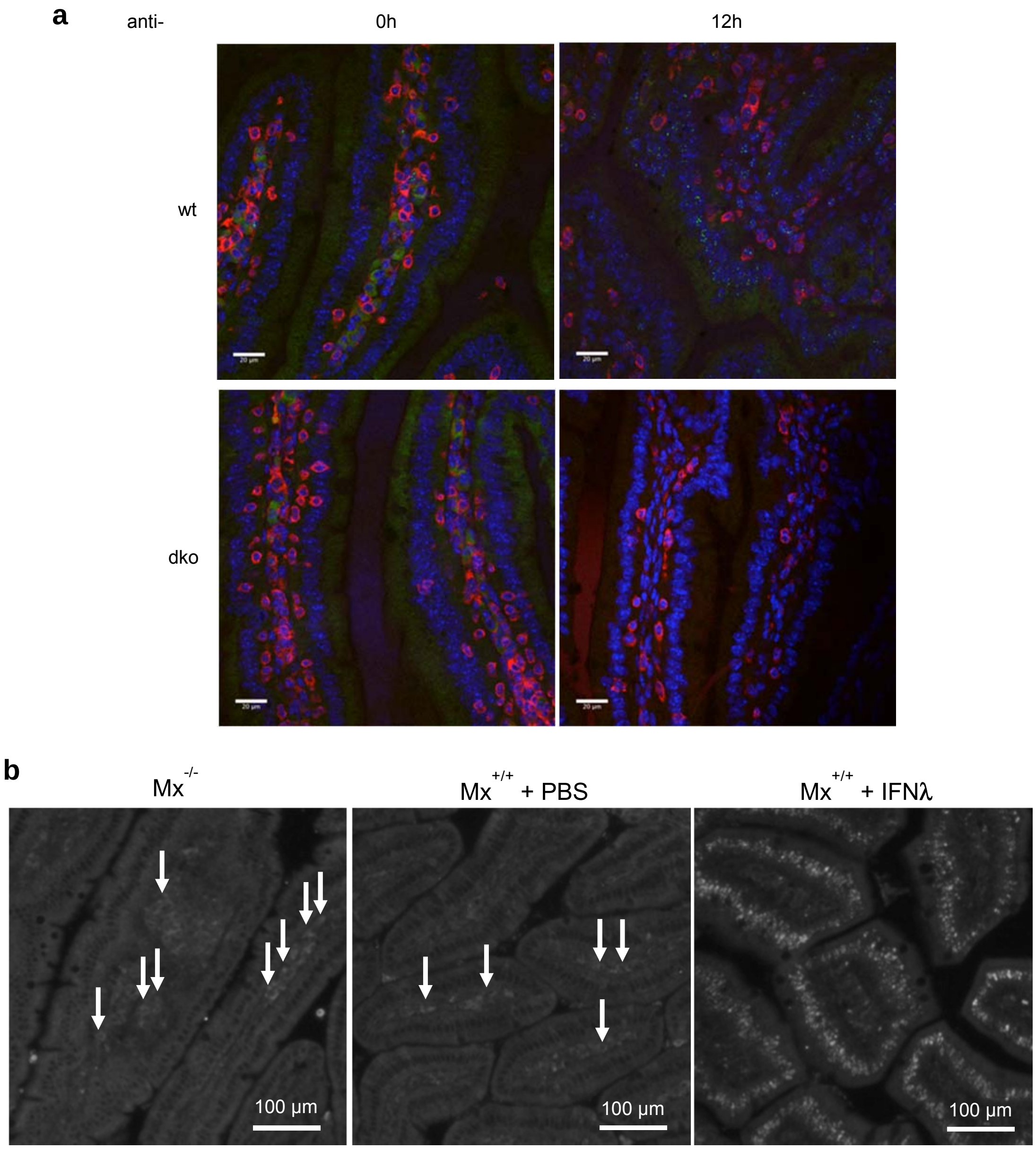

Supplementary Figure 5. Additional data supporting Figure 5. (a) Tissue sections from anti-CD3 i.p. injected B6.A2G-Mx1 wt and IFNAR/IFNLR DKO mice, both of which contain a functional *Mx1* gene, were prepared at 0 and 12 hours after injection. Paraffin-embedded sections were stained for Mx1 protein (intra-nuclear, green), CD3 (red), and nuclei (DAPI, blue). Scale bar=20µm. (b) Staining for Mx1 protein in Mx1-deficient ($Mx^{-/-}$) and Mx1-sufficient ($Mx^{+/+}$) mice treated with PBS, or with IFNλ to induce Mx1 protein expression. Arrows indicate non-specific diffuse cytoplasmic staining seen even in the absence of Mx1 protein expression.

Supplementary figure 6 (to figure 6, Swamy et al)

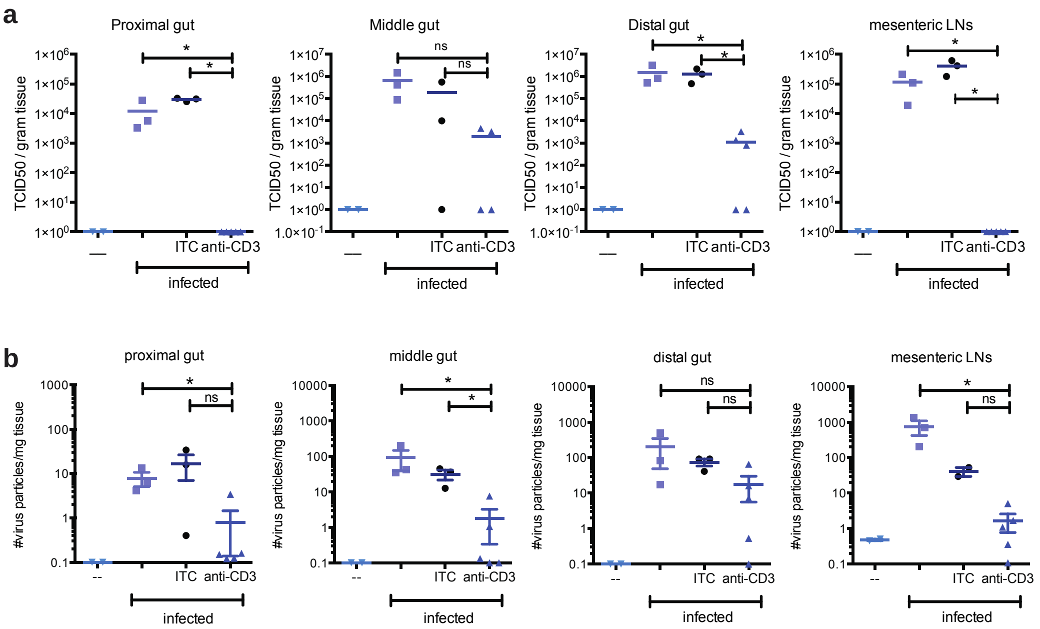

Supplementary Figure 6. Activation of IEL is protective against murine norovirus (MNV) *in vivo*. C57BL/6 mice were orally infected with MNV-O7, without pre-treatment (no label), or 8 hours after treating mice intraperitoneally with anti-CD3 antibodies or the isotype control IgG (ITC). 40 hours after infection, the organs were isolated and assayed for live virus ($TCID_{50}$) per mg of tissue (a) or viral particles (RNA copies) per mg of tissue (b) as in Fig. 6. Uninfected mice were used as controls (--). Statistical significance between conditions was measured by two-tailed Kolmogorov-Smirnov test.

Supplementary Table 1. Sequences of primers used for qRT-PCR.

Gene	Forward primer	Reverse primer	JOE probe
Adar	ggaagaagactcggagaaacc	tcccagagaacaaggatgttg	
Eif2ak2 (Pkr)	ggagcacgaagtacaagcgc	gcaccgggttttgtatcga	
Ifit1	ctccactttcagagccttcg	tgctgagatggactgtgagg	
Ifit2	aaatgtcatgggtactggagtt	atggcaattatcaagtttgtgg	
Ifna (all)	tctgatgcagcaggtggg	agggctctccagacttctgctctg	
Ifnb1	ctggcttccatcatgaacaa	agagggctgtggtggagaa	
Ifng	ttactgccacggcacagtc	agataatctggctctgcagg	
ifnl	agctgcaggccttcaaaaag	tgggagtgaatgtggctcag	
Ifnl3	tcagccctgaccaccatc	ctgtggcctgaagctgtgta	
Il17a	agctccagaaggccctcagactacc	cagctttccctccgcattgacac	
Irf1	gagctgggccattcacac	tccatgtcttgggatctgg	
Irf7	cttcagcactttcttccgaga	tgtagtgtggtgacccttgc	
Mx1	tgtgcaggcactatgaggag	actctggtccccaatgacag	
Oas1g	gcatcaggaggtggagtttg	ggcttcttattgatactaccatgacc	
Oas2	tgcggaagttcctactgacc	cccaccatgtcacttgtcttt	
Tbp	ggggagctgtgatgtgaagt	ccaggaaataattctggctcat	
Tnf	ctgtagcccacgtcgtagc	ttgagatccatgccgttg	
Usp18	ttgggctcctgaggaaacc	cgatgttgtgtaaaccaaccaga	
MNV-Q2	gctttggaacaatggatgctgag	cgctgcgccatcactcatc	ccgcaggaaygctcagcagtctt
